# Identification of Barriers to Access Endovascular Treatment for Acute Ischemic Stroke in the Health Care System of Mexico: Results From a National Survey Among Endovascular Neurologists

**DOI:** 10.3389/fneur.2021.601328

**Published:** 2021-02-09

**Authors:** Fernando Gongora-Rivera, Alejandro Gonzalez-Aquines, Juan Manuel Marquez-Romero, Neurological Endovascular Therapy – Mexican Group (NET-MX Group)

**Affiliations:** ^1^Department of Neurology, Hospital Universitario Dr. José Eleuterio González, Universidad Autónoma de Nuevo León, Monterrey, Mexico; ^2^Department of Neurology, Instituto Mexicano del Seguro Social (IMSS) HGZ 2, Aguascalientes, Mexico

**Keywords:** barriers, endovascular treatment, ischemic stroke, healthcare disparities, developing country

## Abstract

**Background:** Providing endovascular treatment (EVT) access for acute ischemic stroke (AIS) is a challenge in Latin America. Even though the Mexican Endovascular Reperfusion Registry (MERR) and the RESILIENT trial have demonstrated the feasibility of EVT of AIS in Latin America, the MERR has uncovered potential challenges to delivering EVT to AIS patients.

**Aim:** To identify the perceived barriers to access EVT for AIS in Mexico.

**Methods:** We surveyed endovascular neurologists in Mexico. The survey addressed the situation of thrombectomy in the country and the infrastructure and resources available in the participants' institutions. The questionnaire inquired about costs, barriers, and challenges to accessing EVT for AIS, emphasizing the prices and availability of medical devices needed for EVT.

**Results:** We analyzed data from 21 hospitals. The most extreme identified barriers to access EVT were the lack of health coverage for EVT in the National Health System, the cost of the medical supplies for EVT, and inadequate knowledge of stroke symptoms in the general population. The median cost for EVT was USD 20,000 (IQR 7,500–20,000). From this amount, 60% (IQR 50–70%) corresponded to the costs involved with medical devices. EVT carried additional out-of-pocket costs in 90% of the hospitals, and in 57%, the costs exceed USD $10,000.

**Conclusion:** Efforts at all government levels and society are required to tackle these barriers. An increase in and efficient use of public funding for EVT coverage and the deployment of continuous and targeted stroke education campaigns could reduce inequities in EVT access in Mexico.

## Introduction

The burden of stroke has decreased in most countries worldwide in the last few decades (1). A significant portion of this reduction is due to a revolution in managing acute ischemic stroke (AIS) derived from endovascular treatment (EVT). Functional outcomes have improved with EVT. The window of treatment has extended well-beyond the window for intravenous recombinant tissue plasminogen activator (IV t-PA), allowing a higher number of patients to receive treatment that improves their functional independence and quality of life (2).

However, the situation in Latin America is different. The incidence and the number of stroke survivors have increased since 1990 by more than 80 and 90%, respectively (3). Consequently, in 2018, the representatives of health Ministries from 13 Latin American countries committed through the Declaration of Gramado to reduce the burden of stroke in the region by increasing stroke prevention, treatment, and recovery (4). One of the main challenges identified in the Declaration of Gramado is limited access to EVT.

Numerous reports demonstrate the efficacy of EVT for AIS (5) and its cost-effectiveness in reducing acute care, rehabilitation, long-term expenses, and increasing the number of patients reintegrated into their daily life activities (6). However, data from Latin American countries is scarce (7). The Mexican Endovascular Reperfusion Registry (MERR) was the first national multicenter registry of thrombectomy in the region. It showed that EVT for AIS patients is feasible in a developing country (8). Moreover, the registry also uncovered potential challenges to deliver EVT to AIS patients, primarily due to high costs (8). Recently, the RESILIENT trial (9). confirmed the feasibility of EVT of AIS in Latin America in a larger sample of patients. Nevertheless, in the RESILIENT trial, the cost was not an issue since the trial received unrestricted grants for device donations from the device manufacturers.

Derived from the experience with the MERR, the objective of the present study was to identify the perceived barriers to access EVT for AIS in a sample of endovascular neurologists.

## Methods

A nationwide observational, cross-sectional study was performed in April 2020. Participants from the Mexican Endovascular Reperfusion Registry study (8), an academic, independent, prospective, multicentre, observational registry, were invited to complete an online survey regarding the perception of barriers to access EVT for AIS. All participants were endovascular neurologists, which are vascular neurologists with training to perform EVT. They were asked to participate voluntarily and provided informed consent for participation. The survey is available in Supplementary Material 1.

The survey consisted of two sections: (1) the situation of thrombectomy in the country and (2) the infrastructure and resources available in their practicing institutions. The questionnaire inquired about the costs, barriers, and challenges to accessing EVT for AIS, emphasizing the prices and availability of medical devices (catheters, stent retrievers, and, aspiration devices) needed for EVT. To reduce response bias, dichotomous questions were framed neutrally and open-ended questions avoided leading answers. Furthermore, if the participant practiced in both the private and public sectors, we registered information from both settings due to the innate differences between public and private hospitals. Costs of EVT and medical supplies were gathered through a multiple-choice question, and limits were set based on the authors' (FGR and JMMR) experience. When a participant reported more than two practicing institutions, we recorded data from the two most important institutions in terms of the time spent at each institution.

The sample size was calculated by considering a total of 35 hospitals providing EVT in Mexico based on the opinion of EVT specialists due to the lack of available data. Using a confidence level of 90% and a 10% margin for error, the final sample included 24 hospitals. Results are reported as mean (±standard deviation) and numbers with their respective percentages. Barriers were classified on a Likert-type scale ranging from “not a barrier” to “extreme barrier.”

## Results

All Mexican endovascular neurologists, including the MERR collaborators, participated in this survey (response rate 100%), providing information from 21 hospitals. The mean years of practicing EVT for AIS were 9.2 ± 5.2. Only four (26.7%) of the respondents reported working exclusively in public hospitals, while five (33.3%) reported practicing exclusively in private hospitals. Six (40%) provided answers from both settings. Twelve (80%) EVT specialists perceived better access to EVT in the private setting than in public hospitals. The median cost for EVT was USD $20,000 (IQR 7,500–20,000). From this amount, 60% (IQR 50–70%) corresponded to the monetary cost of medical devices.

**Figure 1:**
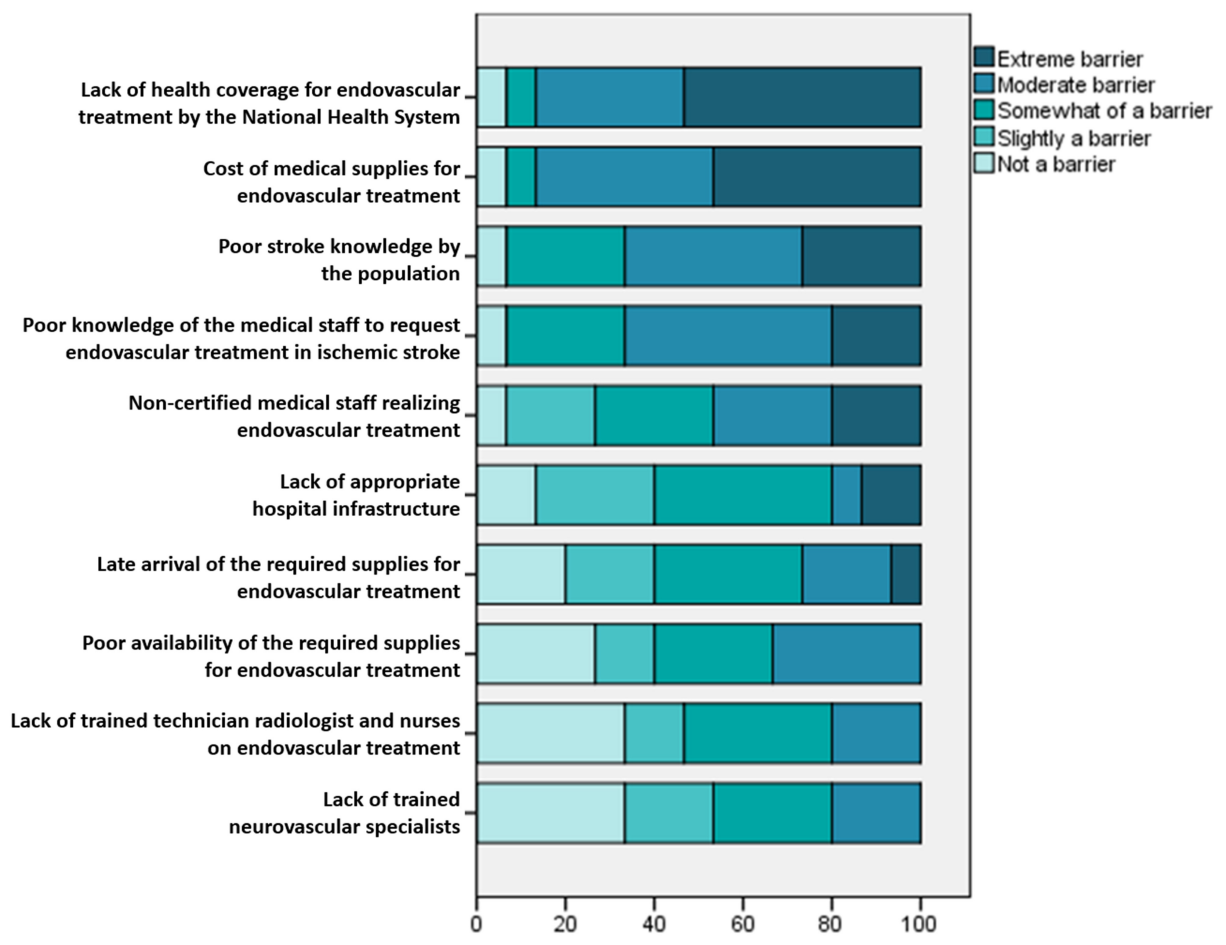
Identified barriers to endovascular treatment in Mexico.

The respondents ranked as the most extreme barrier to access EVT the lack of health coverage for EVT by the National Health System. The cost of medical supplies for EVT was second, followed by inadequate knowledge of stroke symptoms in the general population and the low frequency with which the medical staff request EVT for AIS patients (Figure 1). On the other hand, the participants did not identify as barriers to access EVT: the scarcity of trained endovascular specialists, technician radiologists, and nurses, nor the reduced availability of medical equipment for EVT. We obtained information from 10 public hospitals and 11 private hospitals (Table 1).

**Table 1:** Characteristics of public and private hospitals.

	**Public hospitals *N* = 10**	**Private hospitals *N* = 11**
Geographical location, urban area	10 (100%)	11 (100%)
**Hospital capacity**		
Less than 100 beds	3 (30%)	5 (55.5%)
Between 100 and 500 beds	6 (60%)	6 (54.5%)
More than 500 beds	1 (10%)	0 (0%)
Presence of stroke care unit	5 (50%)	3 (27.3%)
**Access to IV thrombolysis**		
24/7 access	5 (50%)	6 (54.5%)
Limited to certain hours	4 (40%)	0 (0%)
Limited to patients who can afford it	0 (0%)	4 (36.4%)
Not regularly available	1 (10%)	1 (9.1%)
Never available	0 (0%)	0 (0%)
Availability of EVT	6 (60%)	10 (90.9%)
**Access to EVT**		
24/7 access	4 (40%)	3 (27.3%)
Limited to certain hours	0 (0%)	2 (18.2%)
Limited to patients who can afford it	2 (20%)	6 (54.5)
Not regularly available	1 (10%)	0 (0%)
Never available	3 (30%)	0 (0%)
**Access to medical supplies for EVT since requested**		
Immediately (<5 min)	0 (0%)	2 (18.2%)
Less than an hour	6 (60%)	7 (63.6%)
1–24 h	2 (20%)	2 (18.2%)
More than 24 h	2 (20%)	0 (0%)
**Additional out-of-pocket payment compared to IV-tPA**		
No additional cost compared to IV-tPA	2 (20%)	0 (0%)
Less than USD 1,000	0 (0%)	1 (9.1%)
USD 1,000–5,000	2 (20%)	1 (9.1%)
USD 5,001–10,000	3 (30%)	0 (0%)
USD 10,001–20,000	3 (30%)	6 (54.5%)
More than USD 20,000	0 (0%)	3 (27.3%)
**Who covers most of the costs for EVT?**		
Public funding (Local Government)	1 (10%)	0 (0%)
Public funding (National Government)	3 (30%)	0 (0%)
Private insurance	0 (0%)	6 (54.5%)
Patient	6 (60%)	5 (45.5%)
Existence of clinical registry	6 (60%)	5 (45.5%)

*IV-tPA, Intravenous tissue plasminogen activator; EVT, endovascular treatment; USD, United States Dollars*.

The availability of EVT was higher in private hospitals (10 out of 11 hospitals) compared to public hospitals (6 out of 10 hospitals). Regarding the costs of EVT, out-of-pocket payment was substantial; only two out of 10 public hospitals required no additional fee. Overall, in 90% of all hospitals, EVT carries additional out-of-pocket costs. In 57% of the hospitals, these costs exceed USD $10,000.

Three hospitals (one public and in two private) reported 24/7 availability of 1) IV tPA, 2) EVT for AIS, 3) a Stroke Unit, and, 4) EVT devices on-site or available in <60 min after the request. All the other institutions (19 hospitals) lacked one or more components of a comprehensive stroke center.

## Discussion

In this study, we report the barriers to access EVT in a Latin American country. We also describe the cost of EVT for AIS in Mexico. Our results show that EVT is available in public and private settings, but the availability is higher in private hospitals. A comparison between public and private hospitals demonstrated that EVT's greater availability among private hospitals was because the patients being treated in private hospitals could cope with a higher out-of-pocket payment.

The main barrier to accessing EVT in Mexico was the lack of funding to cover the treatment, as perceived by the participants. Despite the high burden of disease that stroke represents in Mexico (10), there is no public funding assigned to the treatment of the disease. In 2017, the Mexican government introduced stroke in the catalog of “catastrophic diseases,” which refers to the conditions that carry a high cost that put those who suffer from it at risk of poverty. However, the designation of stroke as “catastrophic disease” limits funding at the diagnostic stage, covering costs for emergency treatment and diagnostic tests but without covering specific therapies such as IV thrombolysis and EVT (11).

The high cost of the medical supplies for EVT represents the second main barrier to access EVT. In the RESILIENT trial, EVT proved to be a cost-effective intervention for the public sector (8). Nonetheless, the study highlights the relevance of having adequate funding for EVT devices. We consider that since the devices used in the RESILIENT trial were donations from the manufacturers and not paid for out-of-pocket or with public funding, the trial was not representative of a real-world setting (12). The monetary costs of EVT can also express this difference, USD $8,066 vs. > USD $20,000 in our study. These data put pressure on health agencies in Latin American countries to grant public funding for EVT supplies and to increase EVT access.

In our sample, poor knowledge of stroke symptoms in the general population was also a critical barrier to accessing EVT. Poor knowledge of stroke has been reported previously in National reports (13, 14) and is consistent with other Latin American countries (4). A Mexican nationwide study reported that <25% of patients arrived during the first 3 h of stroke onset, with no difference between public and private hospitals (15). Our respondents also identified that the medical staff at their institutions were sometimes unaware of the possibility of EVT or delays in the request for it, contributing to prolonging in-hospital delays to receiving EVT and compromising the patient's outcome (16). The causes of this problem are multiple. They might include logistic difficulties, scarce continuing medical education, and apprehension for the outcome. The creation of multidisciplinary stroke teams inside each institution is a potential solution to reduce in-hospital delays by creating standardized stroke pathways (17). Therefore, it needs to be enforced actively across public and private institutions.

Previous reports show similarities regarding the main barriers for EVT. Tsang et al. (18). assessed the challenges for EVT in developed and developing countries in Asia; their results match ours in treatment cost and triage/diversion system. On the other hand, the need for trained neurointerventionists and comprehensive stroke centers did not appear as significant barriers. A possible explanation for this difference is the heterogeneity in the assessed health systems' size and infrastructure. Since Mexico has three training centers for neurointerventionists and a robust private health system that functions parallel with the government's social security, it is understandable that the perception is similar to that of a developed nation. Nevertheless, as the MERR showed (8), this creates an enormous inequity in EVT access, where only those who can afford the treatment receive care.

According to the Organization for Economic Co-operation and Development (OECD), Mexico has the highest ratio of private hospitals per million inhabitants (28.6) but at the same time has only 11.4 public hospitals per million inhabitants (19). Moreover, the Mexican public health system is fragmented into five different health providers, each operating with its regulations and funding (20). This fragmentation exacerbates the disparity in access to medical care for those in the lower quartiles of income.

Finally, we acknowledge some limitations. Our sample size is small and restricted to endovascular neurologists; nonetheless, we obtained a response rate of all vascular neurologists with an endovascular specialty in México. Our study was realized before the COVID-19 pandemic reached Mexico, and the costs and infrastructure might have changed. Future studies that include the perspectives of endovascular neurosurgeons and neuroradiologists are needed to broaden understanding of limits to access.

## Conclusion

In a sample of endovascular neurologists, we identified three main barriers to EVT: (1) the cost of treatment, (2) the absence of public funding, and (3) poor knowledge of the symptoms of stroke in the general population. Significant efforts at all levels of government and society are required to tackle these barriers. An increase in and the efficient use of public funding for coverage of EVT and the deployment of continuous and targeted stroke education campaigns (for health care professionals and the general population) would create a reduction in the inequities in EVT access in Mexico.

We consider the results of this study as the first step in this direction. By identifying the perceived barriers to accessing EVT, we are in a better position to work with governmental, non-governmental, and private organizations in the development of correcting strategies designed to overcome the current challenges.

## Net-MX Group Members

Alonso Gutiérrez-Romero, Ana Aurora Lugo-Pon, Bernardo Cesar Hernández-Curiel, Claudio Alberto Garcia-Perales, Jose Aurelio Ceron-Morales, Juan Carlos Muñiz-Alvarez, Juan Manuel Santana-López, Luis Manuel Murillo-Bonilla, Marco Antonio Ochoa-Solórzano, Primo Miguel Delgado-Garzón, Ricardo Garcia-Cazarez, Sebastian Gutierrez-Casillas, and Yolanda Aburto-Murrieta.

## Data Availability Statement

The original contributions presented in the study are included in the article/supplementary material, further inquiries can be directed to the corresponding author/s.

## Author Contributions

All authors designed and directed the project, supervised the findings of this work, and co-writing of the manuscript.

## Conflict of Interest

The authors declare that the research was conducted in the absence of any commercial or financial relationships that could be construed as a potential conflict of interest.
