# Supplementary material for: Identification of Barriers to Access Endovascular Treatment for Acute Ischemic Stroke in the Health Care System of Mexico: Results From a National Survey Among Endovascular Neurologists
Source: Front Neurol. 2021 Feb 9;12:601328. doi: 10.3389/fneur.2021.601328 (PMC7900540; doi:10.3389/fneur.2021.601328)
Supplement: Supplementary file 1 [file Data_Sheet_1.PDF]

Supplementary material 1

Questions included in the online survey.	
**Questions related to the National status of thrombectomy**	
In your State, what is the cost (USD) of a standard EVT?	Open ended question
What is the percentage from the total cost of the EVT that corresponds to medical devices needed for EVT (catheters, stent retrievers and, aspiration devices)?	Open ended question
Where do you perceive there is a greater access to EVT?	Likert type question ranked from 1 to 5. 1 means 'the access is grater in public hospitals' and 5 means 'the access is grater in private hospitals.'
How much do you consider the following impede access EVT in your geographical region?<br><br>1. Lack of health coverage for endovascular treatment by the National Health System<br>2. Cost of medical supplies for endovascular treatment<br>3. Poor stroke knowledge by the population<br>4. Poor knowledge of the medical staff to request endovascular treatment in ischemic stroke<br>5. Non-certified medical staff realizing endovascular treatment<br>6. Lack of appropriate hospital infrastructure<br>7. Late arrival of the required supplies for endovascular treatment<br>8. Poor availability of the required supplies for endovascular treatment<br>9. Lack of trained technician radiologist and nurses on endovascular treatment<br>10. Lack of trained neurovascular specialists	Likert type question ranked from 1 to 5. 1 means 'not a barrier' and 5 means 'extreme barrier.'<br>*A response was given to each.*
**Questions related to the characteristics of the participants' practicing institutions**	
In what type of hospital(s) do you spend most of the time?	Multiple choice question: a) public, b) private, c) both.
What is the hospital capacity?	Multiple choice question: a) less than 100 beds, b) between 100 and 500 beds, c) more than 500 beds.
Does this hospital have a stroke unit (exclusive are for stroke patients' care)?	Dichotomous question: a) yes, b) no.

In this hospital, is intravenous thrombolysis accessible for all eligible patients?	Multiple choice question: a) yes, it is accessible 24/7, b) Partly, only if the patient attends the hospital at a specific hour during the day (for example, during working hours or weekdays), c) Partly, only if the patient can afford the cost of the procedure, d) Not regularly, e) Never available.
In this hospital, is EVT available?	Dichotomous question: a) yes, b) no.
In this hospital, is EVT accessible for all eligible patients?	Multiple choice question: a) yes, it is accessible 24/7, b) Partly, only if the patient attends the hospital at a specific hour during the day (for example, during working hours or weekdays), c) Partly, only if the patient can afford the cost of the procedure, d) Not regularly, e) Never available.
How long it takes to receive the medical devices needed for EVT (catheters, stent retrievers and, aspiration devices) after being requested?	Multiple choice question: a) immediately (less than 5 minutes), b) less than an hour, c) 1 – 24 hours, d) more than 24 hours.
What are the average out-of-pocket expenses a patient needs to pay for an EVT?	Multiple choice question: a) no additional cost compared to IV-tPA, b) less than USD 1,000, c) USD 1,000 – 5,000, d) USD 5,001 – 10,000, e) USD 10,001 – 20,000, e) more than USD 20,000.
Who covers most of the costs for EVT?	Multiple choice question: a) public funding (local government), b) public funding (national government), c) private insurance, d) patient.
Does this hospital count with a clinical registry?	Dichotomous question: a) yes, b) no.

EVT: Endovascular treatment, USD: United States Dollars.
